# Socio-economic correlates of quality of life in single and married urban individuals: a Polish case study

**DOI:** 10.1186/s12955-022-01966-2

**Published:** 2022-04-02

**Authors:** Daniel Puciato, Michał Rozpara, Marek Bugdol, Barbara Mróz-Gorgoń

**Affiliations:** 1 Faculty of Finance and Management, WSB University in Wrocław, ul. Fabryczna 29-31, 53-609 Wrocław, Poland; 2 Institute of Sport Sciences, The Jerzy Kukuczka Academy of Physical Education, ul. Mikołowska 72A, 40-065 Katowice, Poland; 3 Faculty of Management and Social Communication, Jagiellonian University, ul. prof. St. Łojasiewicza 4, 30-348 Kraków, Poland; 4 Faculty of Business and Management, Wroclaw University of Economics and Business, ul. Komandorska 118-120, 53-345 Wrocław, Poland

**Keywords:** Quality of life, Socioeconomic status, Marital status

## Abstract

**Background:**

One of key current social trends is the increasing number of single people. It has multiple implications as single individuals often live and behave differently than those living in relationships. Marital status and socioeconomic status may also be significant quality of life factors for single persons. The aim of this study is to identify relationships between quality of life and selected indicators of socioeconomic status in single and married respondents from the Wrocław metropolitan area in Poland.

**Methods:**

4460 respondents took part in the study (1828 single, 2632 married). The study was cross-sectional based on a diagnostic survey. Data was gathered on respondents’ sex, age, education, marital status, occupational status and financial situation as well as their quality of life and perceived health condition. Frequencies (f) and relative frequencies (rf) of categories of dependent and independent variables were determined. The chi-squared test (χ^2^) and odds ratio (OR) statistics were applied. The level of statistical significance was set at α = .05.

**Results:**

A stochastic dependence (p ≤ .05) between marital status and perceived health condition and quality of life in the social domain was found among the respondents. Male sex, higher education, being an entrepreneur, college student or white-collar worker, and good financial status were associated with the highest assessments of quality of life and perceived health condition. The directions of quality of life modifications determined by socioeconomic status were similar in single and married urban respondents; however, the strength of these modifications was greater in the latter.

**Conclusions:**

It is recommended to target respondents with public health programs aimed at lifestyle improvement, tailored to the needs of single and married individuals. Public policies directed at improving education and material situation of respondents are also worth considering, as they may be essential for modeling their quality of life. In addition, research on quality of life should be continued, which is particularly relevant in a pandemic situation.

## Introduction

The issue of quality of life for people of working age is of major research and practical significance. Yet there has been relatively limited previous research on the quality of life of single people. In fact, between 1980 and 2015, the number of single-person households worldwide increased from about 118 million to 300 million. It is expected to rise by another 120 million by 2030. In 2019, single-person households were the most common household type in Europe, accounting for 32.9% of all households in Europe (24.1% in Poland) [1].

Moreover, research results indicate significant differences between single and married people in such matters as consumer behaviors [2], healthcare expenditure [3], or health behaviors [4, 5]. Consequently, it should be assumed that marital status is also relevant for modelling the quality of life of people of working age. The results of previous studies, however, are not conclusive, as they indicate different directions and strengths of relationships between quality of life and marital status. Wahl et al. [6], Kim and Kim [7], and Kowalska et al. [8] observed lower quality of life scores in working-age single individuals compared to married persons. Similar findings have been reported among older adults [9–11] and patients [12, 13]. Emrani et al. [14], Rezaei et al. [15] and Nayir et al. [16], however, reported higher quality of life scores in people living alone than in people in relationships. On the other hand, Raymakers et al. [17] found no significant relationships of quality of life with marital status in their study. Therefore, the problem of quality of life assessment in single people remains open and is addressed in the current study.

Another important issue is the relationship of the quality of life of single people with their socio-economic status. Such a relationship can be assumed to exist as one-person households differ from multi-person households in terms of number of income sources, access to certain social programs, or the size and structure of expenses. Lim's [18] study of U.S. single-person households showed, for example, that single people work and save more than married people of similar socioeconomic status. However, earlier studies on socio-economic determinants of the quality of life of single people of working age have been scarce and concerned only narrow groups of respondents. It was observed that among single people of working age, male gender [19] and younger age [20] may be predictors of higher quality of life. Kim and Kim [7] reported positive associations of quality of life of single mothers with educational level and material situation. Chen et al. [21] and Liu et al. [22], on the other hand, observed that education level and economic status may positively affect the quality of life of single post-working age individuals.

The presented literature review therefore indicates the existence of several research gaps. Firstly, it is still an open research problem whether the quality of life assessment in single people is higher or lower than in married people. Secondly, it is still unclear whether socioeconomic status moderates the quality of life of working-age single individuals. Thirdly, whether socioeconomic modifiers of quality of life are similar for single and married individuals is also an interesting and open research problem. Bridging the indicated research gaps is the key task of the present study.

### Objectives

The aim of this study is to identify relationships of overall quality of life, perceived health condition and health-related quality of life with selected indices of socioeconomic status in single and married adults from the metropolitan area of Wrocław, Poland. The following research questions were addressed in the study:

1. What is the assessment of overall quality of life, health status, and health-related quality of life by single and married respondents?
2. Do sex, age, education level, occupational status, having a steady source of income, per capita income, savings and debt modify respondents’ assessment of their overall quality of life, perceived health condition and health-related quality of life?
3. Are the potential effects of socioeconomic correlates of quality of life similar among single and married respondents?

## Methods

### Study design

**Fig. 1:**
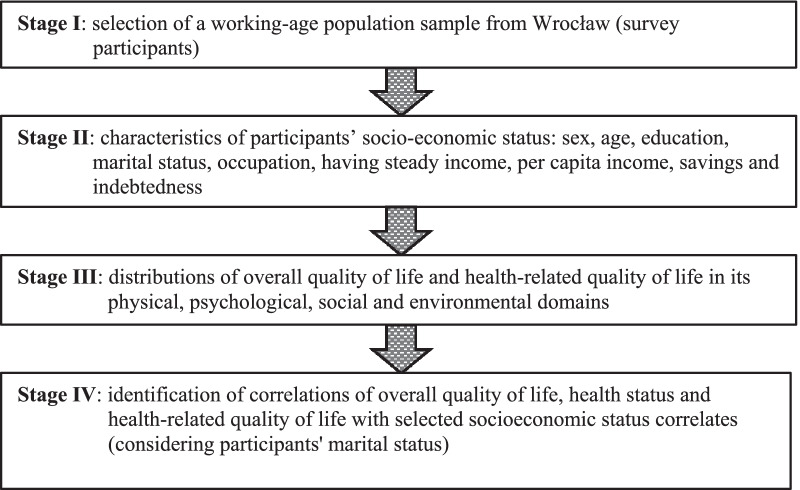
Flowchart of study stages. *Source*: author’s own

The research project had been approved by the Commission of Bioethics of the University School of Physical Education in Wrocław. The study had a cross-sectional survey design. The method of a diagnostic survey with the questionnaire technique was applied. A flowchart of study stages is shown in Fig. 1.

### Sample selection and size

The study was carried out in the Wrocław urban area. Wrocław is a city in south-western Poland, located close to the border with the Czech Republic and Germany, with a population of approximately 638,000. Wrocław is the fastest growing Polish city and has repeatedly been ranked among the top cities in the European Union in socio-economic development rankings. Research results also indicate that Wrocław has some of the highest values of quality of life indices in Poland [23]. These premises were the most important factors behind the choice of Wrocław as a research area.

The study population consisted of 4460 persons (2331 women and 2129 men) of working age 18–64 years living in Wrocław. Among them 1828 people were single, including 1000 women and 828 men, and 2632 were married, including 1331 women and 1301 men. The division of respondents into single and married persons was based on the extended definition of de facto marital status used in European official statistics [24]. The group of single respondents included single, widowed, divorced, separated, and legally separated persons as well as persons not living in a consensual union with another person, legally married persons but not forming a de facto marriage and not living in a consensual union with another person. The group of married respondents included married persons and persons living with their partners.

The sample size was estimated according to the following formula for a finite population [25]:

$$n = \frac{{Nz^{2} p(1 - p)}}{{E^{2} (N - 1) + z^{2} p(1 - {\text{p}})}}$$

where *N*—the number of Wrocław inhabitants as of December 31, 2013 (*N* = 632,067); *p*—the proportion of Wrocław working age population as of December 31, 2013 (*p* = 0.63); *E*—the margin of error (*E* = 0.015); *z*—the z-score associated with a 95% confidence interval (*z* = 1.96).

The sample size was increased by 15% from the estimated sample size to account for potential refusal to participate in the survey.

The selection of the sample was multistage and mixed (random and purposive). In the first stage, using a random number table, 10 housing estates in Wrocław were drawn. In the second stage, using the same random mechanism, 3 streets were selected from each of the 10 housing estates. In the last stage, from among passers-by encountered in the selected streets, every fourth person was asked to participate in the survey.

The inclusion criteria were address of residence in one of the selected streets and working age (18–64 years). The exclusion criteria included chronic diseases, e.g. cancer, diabetes, arterial hypertension, osteoarthritis, osteoporosis. All respondents were informed about the purpose and course of the survey and their voluntary participation. They were asked to provide their informed consent to participate. Out of 4548 individuals who were asked to participate, 88 declined to take part in the survey.

### Measures

#### Socioeconomic background

Data were obtained on the empirical distributions of selected socio-economic indicators, i.e. sex (woman, man), age (under 34 years, 35–44 years, 45 and more years), education (primary, secondary, university), occupation (laborer, white collar worker, entrepreneur, student, unemployed), having steady income (YES, NO), per capita income (under USD 260, USD 260–400, over USD 400), having money savings (YES, NO), and being in debt (YES, NO) as independent variables; and marital status (single, married) as the stratifying variable.

#### WHOQOL

The World Health Organization Quality of Life (WHOQOL BREF) [26, 27] questionnaire was used to assess respondents’ quality of life. It is a tool commonly used in population-based quality of life research. Jaracz et al. [28] and Kowalska et al. [29] demonstrated that the Polish adaptation of the questionnaire is a reliable instrument of quality of life assessment also in adults and economically active people.

The questionnaire consisted of 26 closed questions with answers on a five-level Likert scale. Answers to particular questionnaire items were used in accordance with the accepted data processing key to determine the following indicators: overall quality of life (1–5 pts.); perceived health condition (1–5 pts.); and health-related quality of life in four domains: physical (7–35 pts.), psychological (6–30 pts.), social (3–15 pts.), and environmental (8–40 pts.). Quality of life and perceived health condition indices were converted into an ordinal scale. The 33rd and 66th percentiles were used as division points, where: < 33th percentile indicated low quality of life, 33–66th percentile—average quality of life, and > 66th percentile—high quality of life. In the present study, quality of life and perceived health condition were regarded as dependent variables.

### Statistical analysis

The collected data were subjected to statistical analysis, following which the frequencies (f) and relative frequencies (rf) of categories of considered dependent and independent variables were determined. Pearson's chi-squared test (χ^2^) was used to assess the differences in the distributions of these variables in groups of respondents divided by their marital status [30]. Multinomial logistic regression, including odds ratio (OR) and the confidence interval (CI) [31, 32], was used to assess the relationships between: overall quality of life, perceived health condition and health-related quality of life in the physical, psychological, social and environmental domains, and socioeconomic status, when stratifying respondents by their marital status. The statistical significance of the difference between ORs in the groups of single and married respondents was verified using *z* = *δ/SE(δ),* where: *δ* is the absolute difference between the natural logarithm of OR in each group, and *SE(δ)* is the standard error of this difference [32]. The level of statistical significance was set at α = 0.05. All statistical calculations were made using IBM SPSS Statistics 26 (IBM Corporation, Armonk, NY, USA).

## Results

### Participants

The respondents’ characteristics are shown in Table 1.

**Table 1:** Socio-economic characteristics of respondents (*N* = 4460)

Variable	Total	Single respondents	Married respondents
	*f* (*rf*)	*f* (*rf*)	*f* (*rf*)
*Sex*			
Woman	2331 (52.3)	1000 (54.7)	1331 (50.6)
Man	2129 (47.7)	828 (45.3)	1301 (49.4)
*Age*			
≤ 34 years	1809 (40.6)	1326 (72.5)	483 (18.4)
35–44 years	882 (19.8)	190 (10.4)	692 (26.3)
≥ 45 years	1769 (39.7)	312 (17.1)	1457 (55.4)
*Education*			
University	1103 (24.7)	343 (18.8)	760 (28.9)
Secondary	1617 (36.3)	834 (45.6)	783 (29.7)
Primary	1740 (39.0)	651 (35.6)	1089 (41.4)
*Occupation*			
Laborer	1165 (26.1)	488 (26.7)	677 (25.7)
White collar worker	1356 (30.4)	452 (24.7)	904 (34.3)
Entrepreneur	616 (13.8)	107 (5.9)	509 (19.3)
Student	637 (14.3)	528 (28.9)	109 (4.1)
Unemployed	686 (15.4)	253 (13.8)	433 (16.5)
*Steady income*			
Yes	3608 (80.9)	1318 (72.1)	2290 (87.0)
No	852 (19.1)	510 (27.9)	342 (13.0)
*Per capita income*			
> 400 USD	2112 (47.4)	801 (43.8)	1311 (49.8)
260–400 USD	1133 (25.4)	482 (26.4)	651 (24.7)
< 260 USD	1215 (27.2)	545 (29.8)	670 (25.5)
*Savings*			
Yes	2128 (47.7)	798 (43.7)	1330 (50.5)
No	2332 (52.3)	1030 (56.3)	1302 (49.5)
*Indebtedness*			
Yes	2107 (47.2)	735 (40.2)	1372 (52.1)
No	2353 (52.8)	1093 (59.8)	1260 (47.9)

*f* = frequency; *rf* = relative frequency (in percent)

#### Quality of life and perceived health condition assessment

Table 2 shows the characteristics of the respondents’ quality of life and perceived health condition. The highest percentage of single respondents (47.4%) and married respondents (48.3%) rated their overall quality of life as average. Low overall quality of life was reported by about 36% of respondents regardless of their marital status, while high quality of life was stated by 16.3% of single and 15.3% of married respondents. The single Wrocław residents were significantly more likely (p ≤ 0.05) than married residents to rate their perceived health condition as average (52.8% versus 43.3%) or high (14.7% versus 9.9%). In contrast, 32.4% of single and 46.8% of married respondents rated their perceived health condition as low. There were no significant differences (p > 0.05) in health-related quality of life in the physical, psychological, and environmental domains between single and married respondents. In both groups, the highest percentage of respondents assessed their health-related quality of life as average. On the other hand, the study results revealed a stochastic dependence (p ≤ 0.05) between health-related quality of life in the social domain and marital status. It was rated as average by 40.2% of single and 51.3% of married respondents, and low by 30.3% and 27.4% of respondents, respectively. High quality of life in the social domain was reported by 29.5% of single and 21.4% of married respondents (Table 2).

**Table 2:** Quality of life of respondents (*N* = 4460)

Variable	Total	Single respondents	Married respondents
	*f* (*rf*)	*f* (*rf*)	*f* (*rf*)
*Overall quality of life*			
High	701 (15.7)	298 (16.3)	403 (15.3)
Average	2139 (48.0)	867 (47.4)	1272 (48.3)
Low	1620 (36.3)	663 (36.3)	957 (36.4)
*Perceived health condition*			
High	530 (11.9)	269 (14.7)	261 (9.9)^*^
Average	2106 (47.2)	966 (52.8)	1140 (43.3)
Low	1824 (40.9)	593 (32.4)	1231 (46.8)
*Physical domain of quality of life*			
High	1376 (30.9)	566 (31.0)	810 (30.8)
Average	1840 (41.3)	727 (39.8)	1113 (42.3)
Low	1244 (27.9)	535 (29.3)	709 (26.9)
*Psychological domain of quality of life*			
High	1321 (29.6)	546 (29.9)	775 (29.4)
Average	1768 (39.6)	701 (38.3)	1067 (40.5)
Low	1371 (30.7)	581 (31.8)	790 (30.0)
*Social domain of quality of life*			
High	1103 (24.7)	540 (29.5)	563 (21.4)^*^
Average	2083 (46.7)	734 (40.2)	1349 (51.3)
Low	1274 (28.6)	554 (30.3)	720 (27.4)
*Environmental domain of quality of life*			
High	1244 (27.9)	533 (29.2)	711 (27.0)
Average	1792 (40.2)	714 (39.1)	1078 (41.0)
Low	1424 (31.9)	581 (31.8)	843 (32.0)

*f*, frequency; *rf*, relative frequency in percent

^*^p ≤ .05 for the difference between single and married respondents by chi square test of independence

#### Overall quality of life in terms of socio-economic status

Table 3 presents the results of multinomial logistic regression illustrating the relationships of overall quality of life and perceived health condition (dependent variables) with selected socio-economic status indicators (independent variables), considering the respondents' marital status as the stratifying variable. The odds of high as compared to low overall quality of life were about 35% (OR 0.66, CI 0.50–0.87) lower in single women and 25% (OR 0.75, CI 0.59–0.95) lower in married women than in men. In respondents in the youngest age group, the odds of average versus low overall quality of life were more than four times (OR 4.36, CI 3.26–5.83) higher in single respondents and more than two times (OR 2.02, CI 1.60–2.57) higher in married respondents than in those in the oldest age group. The odds ratio differences in single and married individuals were statistically significant (p ≤ 0.05). Also, the odds of reporting high versus average overall quality of life were more than threefold (OR 3.12) higher in the single respondents and nearly twofold (OR 1.62) higher in married respondents for those aged 35–44 compared to those aged 45 and older. The z-test values also indicated significant differences (p ≤ 0.05) in OR between the groups of respondents of different marital status. The conditional probability of high and not low assessment of overall quality of life was almost three times (OR 2.99, CI 2.05–4.36) higher in the group of respondents with a higher education and almost seven times (OR 6.90, CI 5.12–9.29) higher in the group of married people than in those with a primary education. Also, respondents with a secondary education had higher odds of high versus low overall quality of life scores (OR 1.71 in single respondents, and OR 1.41 in married respondents) than those with a primary education. In both cases, the odds ratio values were significantly different in groups with different marital status (p ≤ 0.05). Occupational status was also a significant modifier of overall quality of life among the respondents. Compared to the reference group, i.e. the unemployed, the highest odds of reporting high rather than low overall quality of life were found among single entrepreneurs (OR 5.05) and married entrepreneurs (OR 48.21), single college students (OR 3.04), married students (OR 16.38), and single white-collar workers (OR 2.08) and married white-collar workers (OR 11.95). All differences in OR values between those single and married respondents were statistically significant (p ≤ 0.05) (Table 3).

**Table 3:** Overall quality of life, perceived health condition with regard to selected socioeconomic indicators in single and married people (*N* = 4460)

IVs	Overall quality of life				Perceived health condition			
	Single	Married	Single	Married	Single	Married	Single	Married
	*OR*_H-L_ (± 95% *CI*)		*OR*_H-L_ (± 95% *CI*)		*OR*_H-L_ (± 95% *CI*)		*OR*_H-L_ (± 95% *CI*)	
*Sex*								
Woman	0.66 (0.50–0.87)	0.75 (0.59–0.95)	0.97 (0.79–1.19)	0.89 (0.75–1.05)	0.51 (0.38–0.68)	0.94 (0.72–1.23)^*^	0.75 (0.61–0.92)	0.91 (0.77–1.07)
Man	1.00	1.00	1.00	1.00	1.00	1.00	1.00	1.00
*Age*								
≤ 34 years	2.89 (1.99–4.19)	2.62 (1.90–3.62)	4.36 (3.26–5.83)	2.02 (1.6–2.57)^*^	3.34 (2.20–5.07)	1.78 (0.95–3.35)	3.23 (2.46–4.23)	2.78 (2.21–3.50)
35–44 years	0.98 (0.52–1.85)	3.00 (2.28–3.93)^*^	3.12 (2.09–4.64)	1.62 (1.32–1.99)^*^	5.24 (3.63–7.56)	5.10 (3.69–7.04)^*^	2.71 (1.83–4.01)	2.17 (1.78–2.65)
≥ 45 years	1.00	1.00	1.00	1.00				
*Education*								
University	2.99 (2.05–4.36)	6.90 (5.12–9.29)^*^	1.21 (0.90–1.63)	3.28 (2.61–4.12)	1.67 (1.11–2.50)	1.12 (0.81–1.55)	1.66 (1.23–2.24)	2.21 (1.82–2.70)
Secondary	1.71 (1.23–2.38)	1.41 (1.03–1.93)^*^	1.39 (1.11–1.74)	1.48 (1.21–1.79)^*^	2.13 (1.53–2.96)	1.82 (1.32–2.52)^*^	1.35 (1.08–1.70)	1.73 (1.42–2.10)
Primary	1.00	1.00	1.00	1.00	1.00	1.00	1.00	1.00
*Occupation*								
Laborer	1.21 (0.75–1.94)	4.25 (2.24–8.07)^*^	1.72 (1.24–2.40)	1.76 (1.37–2.27)	1.62 (0.98–2.67)	13.91 (4.29–45.06)^*^	2.11 (1.52–2.94)	1.92 (1.47–2.51)
White collar worker	2.08 (1.30–3.32)	11.95 (6.46–22.08)^*^	2.39 (1.70–3.36)	3.63 (2.82–4.65)	2.54 (1.56–4.15)	30.28 (9.50–96.52)^*^	2.25 (1.61–3.16)	3.93 (3.05–5.08)^*^
Entrepreneur	5.05 (2.61–9.79)	48.21 (25.58–90.85)^*^	3.79 (2.18–6.58)	5.42 (3.95–7.46)	4.15 (2.12–8.14)	55.34 (17.27–177.31)^*^	2.53 (1.48–4.30)	3.74 (2.80–5.00)
Student	3.04 (1.92–4.81)	16.38 (7.09–37.84)^*^	3.68 (2.62–5.17)	4.87 (2.95–8.04)	2.20 (1.35–3.59)	63.84 (18.01–226.27)^*^	2.63 (1.89–3.65)	4.70 (2.91–7.61)^*^
Unemployed	1.00	1.00	1.00	1.00	1.00	1.00	1.00	1.00
*Steady income*								
Yes	1.52 (1.11–2.08)	9.33 (5.26–16.53)^*^	1.30 (1.04–1.62)	3.57 (2.77–4.59)^*^	1.05 (0.77–1.45)	6.45 (3.47–11.97)^*^	1.16 (0.93–1.46)	5.20 (3.87–6.98)^*^
No	1.00	1.00	1.00	1.00	1.00	1.00	1.00	1.00
*Per capita income*								
> 400 USD	2.82 (2.02–3.94)	16.8 (11.1–25.42)^*^	2.47 (1.94–3.15)	4.68 (3.78–5.79)^*^	4.11 (2.77–6.09)	7.72 (4.88–12.23)^*^	1.44 (1.13–1.82)	2.37 (1.94–2.90)^*^
260–400 USD	1.55 (1.06–2.28)	3.29 (2.03–5.34)^*^	1.80 (1.38–2.35)	2.72 (2.16–3.42)^*^	2.95 (1.88–4.61)	2.43 (1.42–4.14)	1.74 (1.33–2.28)	1.46 (1.17–1.83)
< 260 USD	1.00	1.00	1.00	1.00	1.00	1.00	1.00	1.00
*Savings*								
Yes	5.78 (4.29–7.79)	12.37 (9.27–16.5)^*^	2.35 (1.90–2.92)	4.21 (3.51–5.06)^*^	3.58 (2.65–4.84)	3.05 (2.29–4.06)	1.82 (1.47–2.25)	1.70 (1.45–2.00)
No	1.00	1.00	1.00	1.00	1.00	1.00	1.00	1.00
*Indebtedness*								
Yes	0.28 (0.20–0.38)	0.27 (0.21–0.35)	0.52 (0.42–0.64)	0.58 (0.49–0.69)	0.44 (0.32–0.59)	0.56 (0.43–0.73)	0.43 (0.35–0.53)	0.73 (0.62–0.85)^*^
No	1.00	1.00	1.00	1.00	1.00	1.00	1.00	1.00

IVs, independent variables; *OR*, crude odds ratio; *CI*, confidence interval for *OR*; H–L, high vs. low quality of life; M-L, moderate vs. low quality of life

^*^p ≤ .05 for the *OR* difference between single and married respondents by one sample *z* test

The assessment of the overall quality of life was also significantly modified by economic factors. The odds of high versus low assessment of overall quality of life were more than 50% higher in the single respondents (OR 1.52, CI 1.11–2.08) and more than nine times higher in the married respondents (OR 9.33, CI 5.26–16.53) with a steady income than those without a regular income. The odds of high versus low and average versus low overall quality of life also rose with increasing per capita income. In addition, the respondents with savings had higher odds of high versus low (OR 5.78 for single respondents, OR 12.37 for married respondents) and high versus average (OR 2.35 for single respondents and OR 4.21 for married respondents) overall quality of life scores. The modifying effect of economic factors on respondents' overall quality of life was significantly higher (p ≤ 0.05) in married individuals than in single ones. The odds of high versus low and average versus low overall quality of life assessment were also higher in those without debt than in those with debt (Table 3).

#### Perceived health condition in terms of socio-economic status

Among the single respondents, the odds of a high versus low assessment of perceived health condition were almost twice as low among women than men (OR 0.51, CI 0.38–0.68). The odds of a high versus low assessment of perceived health condition were more than five times higher (OR 5.24 in single individuals and OR 5.10 in married individuals) among the respondents aged 35–44 years, and more than three times lower among those under 34 years of age (OR 3.34), compared to those aged 45 and over. The highest, compared to the control group (the unemployed) odds of high vs. low perceived health condition were found among single entrepreneurs (OR 4.15), and among students living in relationships (OR 63.84). Conversely, the odds of a high versus low perceived health condition assessment were more than twice as high among single labourers (OR 2.54, CI 1.56–4.15) and more than 30 times as high among married workers (OR 30.28, CI 9.50–96.52) than among the unemployed. Also, married blue-collar workers had nearly fourteen times higher odds of high versus low perceived health condition scores compared to the reference group (OR 13.91, CI 4.29–45.06). The odds ratio values were significantly higher (p ≤ 0.05) in married than in single respondents (Table 3).

Having a steady source of income was related to perceived health condition only among married individuals. Respondents with a steady income were more than six times as likely as those without to report high versus low perceived health condition (OR 6.45, CI 3.47–11.97). The odds ratio of having a high versus low perceived health condition was more than four times higher among single respondents (OR 4.11, CI 2.77–6.09) and almost eight times higher among married respondents (OR 7.72, CI 4.88–12.23) than among those with a per capita income of ˃ USD 400 than among those with an income < USD 260. The odds ratio values in the two groups were significantly different (p ≤ 0.05). The odds of high versus low perceived health condition were also higher in those with incomes of USD 260–400 than those with incomes < USD 260 (OR 2.95, CI 1.88–4.61 in single and OR 2.43, CI 1.42–4.14 in married individuals). Respondents with savings were more than three times more likely to have high versus low (OR 3.58 in single and OR 3.05 in married individuals) and nearly twice as likely to have an average versus low (OR 1.82—single and OR 1.70—married) perceived health condition. The conditional probability of high versus low and average versus low perceived health condition was also lower in those with debt than those without debt (Table 3).

#### Health-related quality of life in the physical, psychological, social, and environmental domains with regard to socio-economic status

Tables 4 and 5 present the results of multinomial logistic regression demonstrating the relationships of health-related quality of life in the physical, psychological, social, and environmental domains (dependent variables) with selected factors of socioeconomic status (independent variables), and respondents’ marital status as the stratifying variable.

**Table 4:** Quality of life in the physical and psychological domains and selected socio-economic indices in groups of people of different marital status (*N* = 4460)

IVs	Physical domain of quality of life				Psychological domain of quality of life			
	Single	Married	Single	Married	Single	Married	Single	Married
	*OR*_H-L_ (± 95% *CI*)		*OR*_H-L_ (± 95% *CI*)		*OR*_H-L_ (± 95% *CI*)		*OR*_H-L_ (± 95% *CI*)	
*Sex*								
Woman	0.59 (0.46–0.75)	0.55 (0.45–0.67)	0.66 (0.52–0.82)	0.73 (0.61–0.89)	0.61 (0.48–0.77)	0.59 (0.48–0.72)	0.63 (0.50–0.79)	0.55 (0.46–0.67)
Man	1.00	1.00	1.00	1.00	1.00	1.00	1.00	1.00
*Age*								
≤ 34 years	0.48 (0.34–0.67)	0.95 (0.73–1.24)^*^	0.61 (0.44–0.85)	0.73 (0.57–0.94)	1.37 (1.01–1.86)	1.82 (1.39–2.38)	1.51 (1.13–2.03)	1.11 (0.86–1.43)
35–44 years	0.92 (0.56–1.51)	1.04 (0.82–1.32)	1.12 (0.69–1.81)	0.79 (0.63–0.99)	1.17 (0.72–1.90)	2.24 (1.78–2.83)^*^	2.27 (1.48–3.50)	0.95 (0.75–1.19)^*^
≥ 45 years	1.00	1.00	1.00	1.00	1.00	1.00	1.00	1.00
*Education*								
University	1.27 (0.91–1.78)	2.04 (1.59–2.61)^*^	0.97 (0.69–1.35)	1.01 (0.80–1.28)	2.67 (1.91–3.74)	4.41 (3.42–5.68)^*^	1.90 (1.35–2.69)	2.26 (1.78–2.88)
Secondary	0.66 (0.51–0.87)	1.23 (0.97–1.57)^*^	0.94 (0.73–1.20)	0.86 (0.69–1.07)	0.94 (0.72–1.23)	2.16 (1.68–2.76)^*^	1.64 (1.28–2.08)	1.94 (1.56–2.41)
Primary	1.00	1.00	1.00	1.00	1.00	1.00	1.00	1.00
*Occupation*								
Laborer	1.04 (0.70–1.54)	5.07 (3.50–7.35)^*^	0.96 (0.67–1.39)	1.69 (1.28–2.23)^*^	1.07 (0.74–1.55)	1.83 (1.28–2.61)^*^	2.34 (1.61–3.40)	1.66 (1.27–2.17)
White collar worker	1.28 (0.86–1.92)	7.13 (4.98–10.19)^*^	1.13 (0.78–1.66)	1.80 (1.38–2.36)^*^	1.56 (1.07–2.27)	7.67 (5.46–10.77)^*^	2.92 (1.99–4.29)	3.21 (2.45–4.22)
Entrepreneur	1.46 (0.82–2.6)	4.25 (2.87–6.29)^*^	0.95 (0.53–1.69)	1.86 (1.39–2.50)^*^	3.01 (1.71–5.29)	9.83 (6.68–14.45)^*^	2.61 (1.42–4.82)	4.20 (3.03–5.83)
Student	0.79 (0.54–1.17)	4.03 (2.28–7.12)^*^	0.88 (0.61–1.25)	1.22 (0.74–2.02)	1.50 (1.04–2.15)	4.41 (2.54–7.64)^*^	2.64 (1.81–3.84)	1.83 (1.10–3.05)
Unemployed	1.00	1.00	1.00	1.00	1.00	1.00	1.00	1.00
*Steady income*								
Yes	1.57 (1.2–2.04)	6.12 (4.19–8.93)^*^	1.27 (1.00–1.62)	1.84 (1.44–2.36)^*^	1.71 (1.32–2.21)	4.03 (2.86–5.67)^*^	1.85 (1.45–2.36)	1.84 (1.43–2.37)
No	1.00	1.00	1.00	1.00	1.00	1.00	1.00	1.00
*Per capita income*								
> 400 USD	1.13 (0.86–1.50)	4.05 (3.13–5.25)^*^	1.59 (1.22–2.07)	2.00 (1.59–2.51)	1.67 (1.26–2.20)	4.43 (3.44–5.71)^*^	1.39 (1.07–1.80)	2.33 (1.86–2.91)^*^
260–400 USD	1.43 (1.05–1.95)	1.18 (0.88–1.58)	1.49 (1.10–2.01)	1.16 (0.91–1.48)	1.12 (0.82–1.54)	1.40 (1.04–1.90)	1.35 (1.01–1.80)	1.82 (1.43–2.33)
< 260 USD	1.00	1.00	1.00	1.00	1.00	1.00	1.00	1.00
*Savings*								
Yes	2.40 (1.88–3.07)	2.30 (1.87–2.83)	1.93 (1.53–2.44)	2.02 (1.66–2.45)	2.38 (1.87–3.03)	2.72 (2.22–3.34)	1.55 (1.24–1.95)	1.52 (1.27–1.84)
No	1.00	1.00	1.00	1.00	1.00	1.00	1.00	1.00
*Indebtedness*								
Yes	0.67 (0.52–0.85)	0.57 (0.46–0.69)	0.67 (0.54–0.84)	0.52 (0.43–0.63)	0.53 (0.42–0.67)	0.55 (0.45–0.68)	0.76 (0.61–0.95)	1.00 (0.83–1.20)
No	1.00	1.00	1.00	1.00	1.00	1.00	1.00	1.00

IVs, independent variables; *OR*, crude odds ratio; *CI*, confidence interval for *OR*; H–L, high vs. low quality of life; M-L, moderate vs. low quality of life

^*^p ≤ .05 for the *OR* difference between single and married respondents by one sample *z* test

**Table 5:** Quality of life in the social and environmental domains and selected socio-economic indices in groups of people of different marital status (*N* = 4460)

IVs	Social domain of quality of life				Environmental domain of quality of life			
	Single	Married	Single	Married	Single	Married	Single	Married
	*OR*_H-L_ (± 95% *CI*)		*OR*_H-L_ (± 95% *CI*)		*OR*_H-L_ (± 95% *CI*)		*OR*_H-L_ (± 95% *CI*)	
*Sex*								
Woman	0.82 (0.64–1.04)	0.80 (0.64–1.00)	0.93 (0.75–1.17)	0.74 (0.61–0.88)	0.51 (0.40–0.65)	0.65 (0.53–0.80)	0.70 (0.56–0.88)	0.59 (0.49–0.70)
Man	1.00	1.00	1.00	1.00	1.00	1.00	1.00	1.00
*Age*								
≤ 34 years	2.99 (2.17–4.12)	4.10 (3.03–5.54)	2.65 (1.98–3.55)	1.40 (1.07–1.84)^*^	3.15 (2.22–4.46)	2.12 (1.60–2.81)	1.81 (1.37–2.39)	1.52 (1.18–1.96)
35–44 years	1.37 (0.83–2.26)	2.34 (1.80–3.05)	2.51 (1.66–3.79)	1.11 (0.89–1.38)^*^	3.56 (2.21–5.74)	1.64 (1.30–2.06)^*^	1.57 (1.02–2.42)	0.78 (0.63–0.97)^*^
≥ 45 years	1.00	1.00	1.00	1.00	1.00	1.00	1.00	1.00
*Education*								
University	1.59 (1.14–2.21)	3.02 (2.27–4.00)^*^	1.65 (1.19–2.28)	2.07 (1.64–2.62)^*^	2.96 (2.11–4.16)	3.92 (3.05–5.04)	2.15 (1.53–3.01)	1.58 (1.25–2.00)
Secondary	1.21 (0.93–1.58)	2.00 (1.53–2.61)^*^	1.96 (1.53–2.51)	1.21 (0.97–1.49)^*^	1.42 (1.09–1.86)	1.57 (1.22–2.02)	2.06 (1.62–2.63)	1.31(1.06–1.61)^*^
Primary	1.00	1.00	1.00	1.00	1.00	1.00	1.00	1.00
*Occupation*								
Laborer	0.84 (0.57–1.22)	1.97 (1.37–2.84)^*^	1.06 (0.74–1.53)	1.77 (1.36–2.32)^*^	3.49 (2.19–5.56)	1.24 (0.85–1.79)^*^	1.06 (0.76–1.48)	1.58 (1.21–2.05)
White collar worker	0.86 (0.58–1.29)	2.67 (1.89–3.77)^*^	1.72 (1.19–2.49)	2.16 (1.67–2.80)	4.35 (2.70–7.02)	4.43 (3.16–6.21)	1.70 (1.21–2.40)	2.62 (2.01–3.40)
Businessman	1.13 (0.66–1.95)	4.75 (3.20–7.04)^*^	0.90 (0.51–1.57)	3.23 (2.36–4.42)^*^	10.93 (5.64–21.16)	8.81 (6.11–12.69)	2.06 (1.12–3.77)	1.92 (1.39–2.65)
Student	1.66 (1.14–2.43)	5.21 (2.91–9.31)^*^	1.84 (1.27–2.67)	1.83 (1.07–3.14)	5.69 (3.56–9.09)	6.86 (3.75–12.55)	1.84 (1.31–2.59)	3.26 (1.88–5.66)
Unemployed	1.00	1.00	1.00	1.00	1.00	1.00	1.00	1.00
*Steady income*								
Yes	0.96 (0.74–1.25)	2.11 (1.53–2.89)^*^	1.22 (0.95–1.56)	2.53 (1.96–3.27)^*^	1.96 (1.50–2.56)	2.86 (2.10–3.89)	1.43 (1.13–1.81)	2.70 (2.08–3.52)^*^
No	1.00	1.00	1.00	1.00	1.00	1.00	1.00	1.00
*Per capita income*								
> 400 USD	1.23 (0.93–1.62)	4.88 (3.65–6.53)^*^	1.41 (1.08–1.83)	3.31 (2.66–4.13)^*^	2.82 (2.12–3.73)	10.70 (7.87–14.54)^*^	2.48 (1.90–3.23)	2.64 (2.12–3.29)
260–400 USD	1.20 (0.87–1.65)	2.45 (1.77–3.39)^*^	1.48 (1.10–1.98)	1.77 (1.39–2.26)	1.54 (1.12–2.13)	2.67 (1.90–3.75)^*^	1.96 (1.47–2.61)	1.29 (1.02–1.63)^*^
< 260 USD	1.00	1.00	1.00	1.00	1.00	1.00	1.00	1.00
*Savings*								
Yes	1.53 (1.2–1.94)	4.74 (3.74–6.01)^*^	1.07 (0.86–1.34)	3.65 (3.00–4.44)^*^	3.99 (3.10–5.13)	8.93 (7.08–11.25)^*^	2.19 (1.73–2.77)	2.73 (2.25–3.31)
No	1.00	1.00	1.00	1.00	1.00	1.00	1.00	1.00
*Indebtedness*								
Yes	0.42 (0.33–0.54)	0.37 (0.29–0.46)	0.56 (0.45–0.70)	0.56 (0.47–0.68)	0.26 (0.20–0.34)	0.46 (0.38–0.57)^*^	0.39 (0.31–0.49)	0.66 (0.55–0.79)^*^
No	1.00	1.00	1.00	1.00	1.00	1.00	1.00	1.00

IVs, independent variables; *OR*, crude odds ratio; *CI*, confidence interval for *OR*; H–L, high vs. low quality of life; M-L, moderate vs. low quality of life

^*^p ≤ .05 for the *OR* difference between single and married respondents by one sample *z* test

The odds of a high versus low assessment of health-related quality of life in the physical domain were lower in female respondents than in male respondents, regardless of their marital status. Single respondents from the youngest age group were nearly 50% less likely to have a high versus low health-related quality of life in the physical domain than the oldest respondents (OR 0.48, CI 0.34–0.67). Married Wrocław residents with a college education were more than twice (OR 2.04, CI 1.59–2.61) more likely to have a high versus low assessment of health-related quality of life in the physical domain than those with a primary education. On the other hand, among the single respondents, the conditional probability of a high versus low health-related quality of life was lower among those with a secondary education compared to those with a primary education (OR 0.66, CI 0.51–0.87). Health-related quality of life in the physical domain was further associated with occupational status, but only in married respondents. The conditional probability of high versus low health-related quality of life was more than seven times higher in white-collar workers (OR 7.13), blue-collar workers more than five times higher (OR 5.07), while entrepreneurs and students were more than four times more likely to have high versus low scores (OR 4.25 and OR 4.03), compared to the unemployed. The odds of high versus low health-related quality of life in the physical domain were higher in those with a steady source of income compared to those without it (OR 1.57, CI 1.2–2.04 in single respondents; OR 6.12, CI 4.19–8.93 in married respondents). The differences between the odds ratio values in both groups of respondents were statistically significant (p ≤ 0.05). The odds of high versus low health-related quality of life in the physical domain were more than four times higher (OR 4.05, CI 3.13–5.25) in the highest earning married individuals than in the lowest earning respondents. Higher odds of having a high versus low health-related quality of life, compared with the lowest earners, were also reported by single respondents with per capita incomes between USD 260 and USD 400 (OR 1.43, CI 1.05–1.95). In both study groups, the conditional probability of high versus low and average versus low health-related quality of life scores in the physical domain was furthermore higher in those with savings than those without, and in those not in debt than those in debt (Table 4).

The women, irrespective of their marital status, were characterized by a lower rating of health-related quality of life in the psychological domain than men. Among the married men from Wrocław, the odds of high versus low health-related quality of life in the psychological domain was higher in those aged 35–44 (OR 2.24, CI 1.78–2.83) and under 35 (OR 1.82, CI 1.39–2.38), compared to individuals aged 45 and over. In both single and married respondents, the odds of high versus low and average versus low health-related quality of life in the psychological domain increased with the level of education. Moreover, compared to the unemployed, the highest odds of high versus low rating of this domain of health-related quality of life were found among entrepreneurs (single: OR 3.01; married: OR 9.83), white-collar workers (single: OR 1.56; married: OR 7.67) and students (single: OR 1.50; married: OR 4.41). Statistically significantly higher odds ratio values (p ≤ 0.05) were noted among married persons as compared to single persons. In both groups of respondents, the conditional probability of high vs. low and average vs. low scores for health-related quality of life in the psychological domain was higher in people with a steady income compared to those without it; those with savings vs. those without savings; and those without debt vs. those with debt. The odds of high versus low and average versus low quality of life in this domain also increased with the rise in per capita household income (Table 4).

Among respondents in the youngest age group, the odds of a high versus low and average versus low assessment of health-related quality of life in the social domain were higher than those in the oldest age group. Regardless of respondents' marital status, the conditional probability of a high versus low and average versus low rating of health-related quality of life in the social domain was higher for those with a university education compared to those with a primary education. Among student respondents, regardless of their marital status, there was a higher conditional probability of high versus low rating of health-related quality of life in the social domain, compared to those who were unemployed (OR 1.66, CI 1.14–2.43 in single respondents and OR 5.21, CI 2.91–9.31 in married respondents). In addition, among the married respondents, statistically significantly higher odds of high versus low scores were observed in entrepreneurs (OR 4.75, CI 3.20–7.04), white-collar workers (OR 2.67, CI 1.89–3.77), and laborers (OR 1.97, CI 1.37–2.84) compared to the unemployed. Among the married respondents, having a steady income was also associated with higher odds of high versus low (OR 6.12, CI 4.19–8.93) and average versus low (OR 1.84, CI 1.44–2.36) health-related quality of life scores in the social domain compared to those who did not have one. These odds also increased with the rise in per capita income in the households of married respondents. The odds of high versus low health-related quality of life scores in the social domain were higher in those with savings (single: OR 1.53, CI 1.2–1.94; married: OR 4.74, CI 3.74–6.01), compared to those without savings. Statistically significantly higher odds ratio values were reported by married respondents compared to single respondents (p ≤ 0.05). The odds of high versus low and average versus low health-related quality of life in the social domain were also, regardless of marital status, lower in people with debt compared to those without debt (Table 5).

Female sex was related to lower odds of high versus low and average versus low of health-related quality of life in the environmental domain in all respondents, regardless of their marital status. The conditional probability of high versus low rating of health-related quality of life in the environmental domain was higher among respondents from both marital status groups in those aged up to 44 years compared to those aged 45 years and older. Among single and married respondents, the odds of high versus low and average versus low ratings of health-related quality of life in the environmental domain also increased with an education level. Among the respondents, quality of life in the environmental domain was also significantly modified by their occupational status. Entrepreneurs (single: OR 10.93, CI 5.64–21.16; married: OR 8.81, CI 6.11–12.69), students (single: OR 5.69, CI 3.56–9.09; married: OR 6.86, CI 3.75–12.55) and white-collar workers (single: OR 4.35, CI 2.70–7.02; married: OR 4.43, CI 3.16–6.21). The odds of high versus low health-related quality of life scores in the environmental domain was almost twofold in the group of single respondents (OR 1.96, CI 1.50–2.56) and threefold in the group of married respondents (OR 2.86, CI 2.10–3.89), and higher in those with a steady source of income compared to those without it. The odds also increased with per capita income, regardless of respondents' marital status. The single respondents with savings, compared to those without, were also nearly four times as likely to report high versus low health-related quality of life in the environmental domain (OR 3.99, CI 3.10–5.13) and nine times as likely to report high versus low health-related quality of life if they were married (OR 8.93, CI 7.08–11.25). These odds were also nearly four times lower in the single respondents in debt (OR 0.26) and more than two times lower in married respondents in debt (OR 0.46), compared to the non-indebted respondents. The effect of economic factors on the assessment of health-related quality of life in the environmental domain was significantly (p ≤ 0.05) greater in the married respondents than in the single respondents (Table 5).

## Discussion

The study attempted to address three main research questions. The first concerned the assessment of quality of life of single respondents as compared to married respondents. The study results revealed higher assessment levels of perceived health condition and quality of life in the social domain among the single adults. However, in the case of other quality of life correlates, there were no significant differences between the single and married respondents.

The results of previous studies of relationships of quality of life with marital status have been inconclusive. Wang et al. [33] reported higher overall quality of life, perceived health condition, and health-related quality of life assessments in married Chinese men aged 28–65 years, compared to single men. On the other hand, Kim and Kim [7] found higher quality of life levels in married mothers than in single mothers. Kowalska et al. [8] in her analysis of health-related quality of life in the environmental domain in adults from the Upper Silesia region of Poland observed that married respondents were characterized, on average, by a higher assessment of this domain, than single respondents. Also Wahl et al. [6] and Sarla et al. [11] noted a positive effect of relationships on quality of life. Researchers emphasize, however, that it is not so much the fact of mere being in a relationship that is important in the context of modeling quality of life, but its quality, such as the strength of the relationship or the possibility of receiving necessary support. The indicated attributes of relationship quality can positively affect health and well-being, moderate the negative effects of stress, and be strong determinants of mental health improvement and happy living [34, 35]. However, some studies [36] also suggest that social relationships may negatively affect individuals because they may generate pressure, conflict, frustration, or ineffective support. It should also be emphasized that high quality of interpersonal relationships and the possibility of social support do not have to be experienced only by married individuals, but may also apply to those who are single and have good relationships with family, friends or acquaintances. Similarly, authors of some previous studies [14, 15] reported higher overall quality of life assessments among single individuals than married individuals. Moreover, higher health-related quality of life in the physical and environmental domains in single respondents from Turkey, compared to married respondents, was reported by Nayir et al. [16]. On the other hand, Raymakers et al. [17] found no significant associations of quality of life with marital status. The issue of the relationship between quality of life assessment and marital status, therefore, remains open.

The second research problem addressed in the study was the relationship of quality of life of single people with their socioeconomic status. Respondents from the working-age population of Wrocław indicated that such factors as sex, level of education, occupational status, and financial situation significantly affect their quality of life.

The present study has revealed that women exhibited lower quality of life scores than men. The available literature tends to confirm this observation. Higher quality of life scores for men, compared to women, were also observed in Iranian [14], South Korean [37, 38], and Croatian [39] populations. In addition, Nayir et al. [16] reported higher health-related quality of life levels in the physical, psychological, social, and environmental domains among Turkish men compared to women. Aghamolaei et al. [40], on the other hand, after considering the potential impact of other socio-demographic factors, demonstrated that female sex may be independently associated with lower health-related quality of life in the physical and psychological domains. Kowalska et al. [8] also noted a similar effect with respect to health-related quality of life in the environmental domain. Only Wahl et al. [6] in their study on adult residents of Norway observed that women rated their quality of life higher than men.

In the case of the Wrocław respondents, positive correlations between quality of life and level of education were also noted. Similar consistencies were also reported by Wang et al. [33], Rezaei et al. [15], Wahl et al. [6], Han et al. [41], Song et al. [38] and Emrani et al. [14]. Nayir et al. [16] reported an increase in health-related quality of life in the physical, psychological, social, and environmental domains as the level of education rose. A similar observation regarding health-related quality of life in the environmental domain was made by Kowalska et al. [8]. The reason for the noted patterns may be, on average, higher health awareness in better educated individuals, which has been empirically documented [42]. Well-educated people are more likely than less-educated people to implement the so-called pro-healthy lifestyle by undertaking physical activity, eating rationally, or avoiding risky health behaviors [43]. Higher levels of optimism were also found in well-educated individuals [44], which may again be important for quality of life evaluation.

The quality of life of working-age respondents was most strongly influenced by their occupational status. Entrepreneurs, students and white-collar workers rated their quality of life as the highest, while the unemployed assessed it the lowest. Some previous studies also indicated a high quality of life for entrepreneurs compared to representatives of other occupational groups [45–47]. The main reasons for the entrepreneurs' high quality of life include good material situation [48], opportunity for development [46] and financial independence [49]. On the other hand, higher values of the health-related quality of life in the environmental domain in white-collar workers, than in blue-collar workers, were reported by Kowalska et al. [8]. In a study of an Iranian population, Emrani et al. [14] found significantly higher quality of life levels in students and employed people, compared to the unemployed and housewives. Higher health-related quality of life in the physical and psychological domains among the employed Turks, compared to the unemployed, was also observed by Nayir et al. [16]. Also, Wahl et al. [6] noticed the lowest quality of life measures in unemployed respondents. In addition, Norstrom et al. [50] documented a deterioration in the quality of life and perceived health condition of jobless Swedish adults.

The results of the present study also reveal a positive relationship between quality of life and financial situation. Similarly, in the study by Wang et al. [33], the respondents' quality of life was significantly and positively associated with the level of annual income. An increase in the values of health-related quality of life with the improvement of material situation, mainly the income level, was also reported by Nayir et al. [16], Kulik et al. [51], Rėklaitienė et al. [52], Kooi-Yau Chean et al. [53], Povey et al. [54], Han et al. [41], Huang et al. [55] and Song et al. [38]. The highest quality of life assessment in people with the highest income was found by Rezaei et al. [17], while the lowest quality of life assessment in people with the lowest income by Zhang et al. [56]. Yasartürk et al. [57] reported positive correlations between quality of life and leisure satisfaction and personal and family income in college students. Whereas the significant relevance of financial security, including family income and medical insurance coverage, to health-related quality of life was also confirmed by Chiu and Yang [58]. The study of the Greek population by Kokaliari [59] provides interesting insights. The author showed that for modeling the quality of life, not only the household material situation is important, but also the state of the national economy. He also found that the quality of life of people deteriorates significantly, for example, due to the incidence of economic crises. This is supported by Wong [60] who found that the economic deterioration of a country and the stress associated with it are almost always associated with lower quality of life and sometimes even with an increased mortality rate in citizens. Some authors also indicate particularly strong, positive relationships between quality of life and income situation in countries with a medium level of economic development, which also includes Poland. Frijters et al. [61] demonstrated, for example, that higher real household income levels resulted in a significant increase in life satisfaction among the residents of East Germany following reunification.

The third research problem explored in this study was whether socioeconomic modifiers of quality of life were similar in single and married respondents. Among the Wrocław residents of working age, the relationships between quality of life and socio-economic factors were similar among people of different marital status, but the strength of their impact was higher in married than in single respondents. The differences were particularly significant with respect to education, occupational status, and financial situation. This research problem has not been addressed in previous studies. However, Asakawa et al. [62] in their analysis of a Canadian population noted that socioeconomic variables such as education and material status were important predictors of quality of life scores in working-age individuals. However, at later ages, lifestyle factors become more important than socioeconomic status in older adults. This observation confirms the relationships between quality of life and socioeconomic factors in working-age individuals found in the present study. It can also be assumed the lifestyle played a more important role in shaping the quality of life in single people than any other socioeconomic factors. For example, the lack of a steady source of income does not have to have such a strong negative impact on quality of life as it does in the case of people with families with children. In addition, single persons have more leisure time on average than those in relationships. They can also allocate some of this surplus leisure time for health behaviors such as undertaking physical activity, following a rational and balanced diet, or for preventive health care. Higher levels of health behaviors in single individuals compared to those in relationships have already been documented [63]. Engaging in such health behaviors may directly and positively affect one's health status and, consequently, quality of life, which was reflected in the present study. In addition, the unmarried respondents from Wrocław were also on average younger than those in relationships, which may also have some relevance to their health status and quality of life, since, for example, the level of physical activity often decreases with age [43].

### Study strengths and limitations

The article has its strengths and limitations. One strength is the assessment of socioeconomic modifiers of quality of life, conducted separately for single and married individuals. This is particularly important as the study reports significant differences in the strength of the modeling of quality of life by socioeconomic factors in both groups of respondents. It is also noteworthy that the survey was conducted among healthy individuals of working age, as previous studies have focused on the elderly and sick. The study also includes a research sample representative for the population of the Wrocław metropolitan area. Potential modifiers of quality of life such as having a steady source of income, income per capita, savings or debt have not been considered so far. The limitation of the study is its spatial scope as it was confined to one city only. This makes it impossible to apply the results of the study to the general population. Future research should consider a research group representative for Poland as a whole and even for other Central and Eastern European countries. Cross-sectional surveys should also be replaced by more continuous surveys, e.g. cohort-based, in order to take into account the effects of changes in marital status on quality of life. The dichotomous division of respondents into single and married ones is another limitation. Future research should consider subcategories of single respondents, i.e.: unmarried, widows and widowers, divorced, as well as of married respondents, e.g. married and in partnerships. An interesting research question in the context of the quality of life of single people may also be whether living alone is the result of a conscious, voluntary choice or results from the life situation. Similarly, with regard to people in relationships, future research should consider an assessment of the quality of those relationships.

## Conclusions

The findings of the study permit the formulation of three research conclusions:

1. Among the Wrocław respondents of working age, the single adults on average rated their health status and quality of life in the social domain higher than married adults.
2. Male gender, higher education, being an entrepreneur, student, or white-collar worker, and good financial situation were associated with the highest ratings of quality of life among single respondents.
3. The directions of quality of life modeled by socioeconomic status were similar in both groups of respondents, but the strength of the socioeconomic status impact was greater in married individuals than in single individuals.

The findings of the study have some practical implications. First, despite the respondents' generally good assessment of their quality of life, about one-third of the single and almost half of the married respondents rated their health poorly. It is therefore appropriate to recommend targeting respondents from both groups with public health programs aimed at improving their lifestyles. In addition, these programs should be tailored to the needs of their recipients, as, for example, the preferred forms of physical activity by single and married individuals may differ. The second practical implication to be formulated in response to the research findings concerns the respondents’ socio-economic determinants of quality of life. It is postulated that economic and social policies aimed at improving the educational level and material condition of society may have a role in modeling the quality of life of respondents, particularly strong in the case of those in married relationships. However, the implementation of such policies is often associated with the problem of coordination of activities aimed at quality of life improvement carried out by various bodies, such as state administration, local government, or businesses. In this context, it is important to integrate these activities and support them in accordance with the principles of public management, following the conclusions of quality of life diagnosis. This is the rationale for the third practical recommendation, since the results of the present study also confirm a need for more detailed assessment of different life situations of single and married adults. In order to improve their quality of life, further diagnostic research is essential, which can be used in social policy programmes covering differences between single and married adults in terms of quality of life domains but also behavioural problems (e.g. the impact of extra responsibilities resulting from raising children or caring for the elderly) and the effects of socioeconomic variables, health status, disability, and employment on quality of life. Further in-depth research on how quality of life is affected by the pandemic situation is also important. In this area, research findings regarding quality of life in the workplace (as this topic has been studied mainly in terms of membership in an organization) and quality of life in relation to interpersonal relationships are crucial for social policy programs.

## Data Availability

The datasets used and/or analysed in the current study are available from the corresponding author on reasonable request.

## Abbreviation

WHOQOL: The World Health Organization Quality of Life
