# Correction to: Socio-economic correlates of quality of life in single and married urban individuals: a Polish case study

**DOI:** 10.1186/s12955-022-01984-0

**Published:** 2022-05-03

**Authors:** Daniel Puciato, Michał Rozpara, Marek Bugdol, Barbara Mróz-Gorgoń

**Affiliations:** 1 Faculty of Finance and Management, WSB University in Wrocław, ul. Fabryczna 29-31, 53-609 Wrocław, Poland; 2 Institute of Sport Sciences, The Jerzy Kukuczka Academy of Physical Education, ul. Mikołowska 72A, 40-065 Katowice, Poland; 3 Faculty of Management and Social Communication, Jagiellonian University, ul. prof. St. Łojasiewicza 4, 30-348 Kraków, Poland; 4 Faculty of Business and Management, Wroclaw University of Economics and Business, ul. Komandorska 118-120, 53-345, Wrocław, Poland

## Correction to: Health and Quality of Life Outcomes (2022) 20:58 https://doi.org/10.1186/s12955-022-01966-2

The authors of the original article [1] mistakenly omitted a funding source which has since been re-introduced.
